# Protective Effects of Nuciferine in Middle Cerebral Artery Occlusion Rats Based on Transcriptomics

**DOI:** 10.3390/brainsci12050572

**Published:** 2022-04-28

**Authors:** Chang Chen, Quantao Ma, Jinzhu Jiang, Tieshan Wang, Linghui Qiu, An Liu

**Affiliations:** 1 Institute of Chinese Materia Medica, China Academy of Chinese Medical Sciences, Beijing 100700, China; cchen@icmm.ac.cn (C.C.); jzjiang@icmm.ac.cn (J.J.); 2 School of Traditional Chinese Medicine, Beijing University of Chinese Medicine, Beijing 100029, China; maquantao@bucm.edu.cn; 3 Beijing Research Institute of Chinese Medicine, Beijing University of Chinese Medicine, Beijing 100029, China; tieshanwang@bucm.edu.cn; 4 School of Acupuncture-Moxibustion and Tuina, Beijing University of Chinese Medicine, Beijing 100029, China

**Keywords:** MCAO, transcriptomic, RNA sequencing, miRNA, circRNA

## Abstract

Middle cerebral artery occlusion (MCAO), with the characteristics of high morbidity, high recurrence rate, high mortality, and disability rate, is a typical manifestation of ischemic stroke and has become a hot research topic in the clinical field. The protective effects of nuciferine on brain injury MCAO rats were investigated and its mechanisms of actions were revealed. The MCAO rats were established by the suture method. The pathological staining of the rat brain was processed and observed, the pharmacodynamics assay of nuciferine were studied, and the gene expression regulation by nuciferine was detected by transcriptome technology. The results showed that nuciferine significantly alleviated brain damage in MCAO rats, and the transcriptomic results suggested that nuciferine could exert therapeutic effects through the regulation of lipid metabolism, including arachidonic acid metabolism, sphingolipid metabolism, the PPAR signaling pathway and other related pathways. This finding provided new perspectives on the treatment of MCAO with nuciferine and facilitates the development of novel drugs for this disease.

## 1. Introduction

Stroke is brain damage caused by cerebral ischemia and hypoxia, which has the characteristics of high morbidity, high recurrence rate, high mortality, and high rate of disability. It mainly includes ischemic stroke, accounting for about 87%, and hemorrhagic stroke [1]. Strokes mainly occur in the elderly, but due to the ageing of society and the irregular lifestyle of young people, the incidence of stroke is increasing year by year and young people are more prone to this disease, which causes worldwide concern. Ischemic stroke and its sequelae seriously affect patients’ life quality and bring a heavy economic burden to families and society [2].

Middle cerebral artery occlusion (MCAO) is a typical example of ischemic stroke and has become a hot topic in research. The brain damage mechanisms of it are complex, and it has been found that some mechanisms associated with MCAO injury had already been reported in published research cases, mainly including disorder of brain energy metabolism, disorder of lipid metabolism [3], atrial fibrillation [4,5], intracellular calcium overload [6,7,8], acidosis [9], oxidative stress [10,11,12,13], stress nitride [14], inflammatory response [15,16], excitatory amino acids toxicity [17], mitochondrial dysfunction and apoptosis [18], and other mechanisms that remain obscure. However, the specific mechanisms are expected to be thoroughly determined in the future.

Nuciferine is an aporphine alkaloid from the dried leaves of the water lily plant, *Lotus Vulgaris*, which is also a quality control component of lotus leaves in the Chinese Pharmacopoeia. Studies have shown that nuciferine has various pharmacological effects such as hypolipidemic, hypoglycemic, antitumour and anti-ischemic effects [19]. It was shown that pre-stroke statin therapy can improve the in-hospital prognosis of patients with atrial fibrillation, especially for acute chemical stroke [20]. The pharmacological effects of nuciferine in regulating lipid metabolism, and whether it can exert therapeutic effects on lipid metabolism disorders caused by MCAO need to be further investigated.

Transcriptomics is a technology that has been developed after genomics, which has become an effective means to study pathological and drug treatment mechanisms in recent years. Furthermore, it reveals gene expression information through high-throughput sequencing enabling the interpretation of the development of disease and drug treatment mechanisms at the genetic level. In this article, we observed the therapeutic effects of nuciferine in rats with MCAO and revealed the therapeutic mechanism of nuciferine at the genetic level through transcriptomics technology, providing a theoretical basis for MCAO treatment.

## 2. Materials and Methods

### 2.1. Chemicals and Reagents

Nuciferine was purchased from Shanghai yuanye Bio-Technology Company, Ltd. (purity ≥ 98%; Shanghai, China). Carboxymethylcellulose sodium (CMC-Na) was purchased from Beijing BioDee Biotechnology Company, Ltd. (Beijing, China). 2,3,5-Triphenyl-2H-Tetrazolium Chloride (TTC solution, 2%) was obtained from Beijing Solarbio Science and Technology Company, Ltd. (Beijing, China). Nissl staining kit was purchased from Beijing Beyotime Biotechnology Company, Ltd. (Beijing, China). Double distilled water was provided by a Milli-Q system (Millipore, MA, USA).

### 2.2. Animals

All animal experiments were performed on male Sprague Dawley rats weighing 240–270 g and were obtained from Beijing Vital River Company (Beijing, China). All rats were housed individually at 25 ± 2 °C, relative humidity of 45 ± 15% with a 12 h light/dark cycle and free access to chow and water. They were allowed to adapt to the housing conditions for two days before the experiments were performed and taken care of by the China Academy of Chinese Medical Sciences’ Laboratory Animal Care Center. All animal experiments were performed following institutional guidelines and ethics.

### 2.3. Animal Models and Experimental Protocol

The rats were anesthetised with sodium pentobarbital (40 mg·kg^−1^ i.p.) after 2 days’ acclimatization. The MCAO operation was performed by intraluminal filament method [21]. The rectal temperature was recorded and maintained at 37 ± 0.5 °C throughout the surgical procedure. Briefly, a fishing thread (diameter of 0.26 mm) with a round tip was inserted from the left common carotid artery into the lumen of the internal carotid artery to occlude the origin of the middle cerebral artery. The rats were randomly divided into the following 3 groups: sham group, vehicle group, and nuciferine-treated group (40 mg·kg^−1^). Nuciferine powders were dissolved in sterile saline (containing 0.5% CMC-Na) to make the stock solution. The sham and vehicle groups were fed an equivalent volume of 0.5% CMC-Na water solution. All rats were sacrificed 24 h after the MCAO.

### 2.4. Assessment of Neurological Defects

In order to reveal the treating mechanism of nuciferine on nerve defects caused by MCAO surgery, the nerve defects of rats were assessed by observing their behavioural performance in conjunction with uniform rules for scoring neurological function. The researchers measured the nerve defects 24 h after MCAO surgery by blind evaluation. The neurological scores were defined as follows: 0 for no neurological deficit; 1 for failure to extend the right forelimb; 2 for circling to the contralateral side; 3 for falling to the contralateral side at rest; and 4 for no spontaneous motor activity.

### 2.5. Evaluation of Cerebral Infarct Volume

After the neurological function scoring was completed, the rats were anesthetised, and brain tissues were collected and washed with physiological saline solution. The cerebral infarct volumes were measured with TTC staining and used for describing the severity of the cerebral ischemia. The intact brain tissues were stored in a flat dish at −20 °C for about 15 min. Then, the brains were sliced into six coronal sections 2 mm thick. The brain slices were treated with a 2% TTC saline solution and incubated at 37.5 °C for 30 min. After staining with TTC, the normal tissue stained a rose-red color, and the infarct tissue was white. The brain slices were photographed using a digital camera under a fixed light source and background. Statistical analysis was performed for infarcted areas in the brain sections using image analysis software (Image-Pro Plus 6.0, Rockville, MD, USA).

### 2.6. Evaluation of Cerebral Edema

Following decapitation at 24 h after MCAO, the left and right cerebral hemispheres were obtained and immediately weighed to obtain the wet weight. The rate of cerebral edema was calculated as the following formula and the infarction cerebral hemisphere was the left brain.

Rate of cerebral edema = (left brain weight − right brain weight)/whole cerebral weight × 100%

### 2.7. Histological Observation

Hematoxylin and eosin (HE) staining was used to detect the pathological changes in the ischemic penumbra 24 h after MCAO (*n* = 6). Briefly, the rats were anesthetised as above, and the brains were fixed via transcranial perfusion with 0.9% cold heparinised saline and 4% paraformaldehyde. The brain was removed and postfixed in 4% (*w*/*v*) paraformaldehyde for 6–8 h. The brain blocks were embedded in paraffin and cut into 5 μm coronal sections. Then, the sections were stained with HE, and the number of HE-positive cells in the ischemic penumbra was counted in 5 different fields for each section in a blind manner by light microscopy (BX51; Olympus, Tokyo, Japan). Data from six sections in each group were averaged.

### 2.8. Nissl Staining

Nissl staining was applied to observe neuronal morphologic changes in the ischemic penumbra 24 h after MCAO operation (*n* = 6). The experimental steps were strictly performed according to the manufacturer’s manual of the Nissl staining kit. An optical microscope was used to observe each slice. The total number of Nissl-positive neurons in the penumbra were counted in six different fields of view for each section by an observer blinded to the treatment group. Nissl bodies in the penumbral area were counted with Image-Pro Plus 6.0.

### 2.9. RNA Extraction

According to the manufacturer’s instructions (Invitrogen, Carlsbad, CA, USA), total RNA was extracted from the cerebral cortex tissue using TRIzol^®^ Reagent according to the manufacturer’s instructions (Invitrogen, Carlsbad, CA, USA), and genomic DNA was removed using DNase I (TaKara, Dalian, China). RNA quality was determined by 2100 Bioanalyser (Agilent, Santa Clara, CA, USA) and quantified using the ND-2000 (NanoDrop Technologies, Wilmington, DE, USA). Only a high-quality RNA sample (OD260/280 = 1.8~2.2, OD260/230 ≥ 2.0, RIN ≥ 6.5, 28S:18S ≥ 1.0, >2 μg) was used to construct a sequencing library.

### 2.10. Library Preparation, and Illumina Novaseq 6000 Sequencing

RNA purification, reverse transcription, library construction and sequencing were performed at Shanghai Majorbio Bio-pharm Biotechnology Company, Ltd. (Shanghai, China) according to the manufacturer’s instructions (Illumina, San Diego, CA, USA). An RNA-seq transcriptome library was prepared following the TruSeqTM RNA sample preparation Kit from Illumina (San Diego, CA, USA) using 1μg of total RNA. Shortly after, messenger RNA was isolated according to the polyA selection method by oligo (dT) beads and then fragmented by fragmentation buffer firstly. Secondly, double-stranded cDNA was synthesized using a SuperScript double-stranded cDNA synthesis kit (Invitrogen, Carlsbad, CA, USA) with random hexamer primers (Illumina). Then the synthesized cDNA was subjected to end-repair, phosphorylation and ‘A’ base addition according to Illumina’s library construction protocol. Libraries were sized for 200–300 bp cDNA target fragments on 2% Low Range Ultra Agarose, followed by PCR amplified using Phusion DNA polymerase (NEB) for 15 PCR cycles. After being quantified by TBS380, the paired-end RNA-seq sequencing library was sequenced with the Illumina Novaseq 6000 (2 × 150 bp read length).

### 2.11. Read Mapping

The raw paired-end reads were trimmed, and quality controlled by SeqPrep (https://github.com/jstjohn/SeqPrep accessed on 25 March 2021) and Sickle (https://github.com/najoshi/sickle accessed on 25 March 2021) with default parameters. Then clean reads were separately aligned to the reference genome using the orientation mode of the TopHat (http://tophat.cbcb.umd.edu/ (accessed on 2 April 2021), version 2.1.1) [22] software. The mapping criteria of bowtie was as follows: sequencing reads were uniquely matched to the genome allowing up to two mismatches, without insertions or deletions. Then the gene region was expanded following the depths of sites, and the operon was obtained. In addition, the whole genome was split into multiple 15 kb windows that share 5 kb. New transcribed regions were defined as more than two consecutive windows without an overlapped gene region, where at least two reads were mapped per window in the same orientation.

### 2.12. Differential Expression Analysis and Functional Enrichment

To identify DEGs (differential expression genes) between two different samples, the expression level of each transcript was calculated according to the fragments per kilobase of exon per million mapped reads (FPKM) method. RSEM (http://deweylab.biostat.wisc.edu/rsem/ accessed on 26 May 2021) [23] was used to quantify gene abundances. R statistical package software EdgeR (Empirical analysis of Digital Gene Expression in R, http://www.bioconductor.org/packages/2.12/bioc/html/edgeR.html accessed on 29 May 2021) [24] was utilised for differential expression analysis. In addition, functional-enrichment analysis including gene ontology (GO) and kyoto encyclopedia of genes and genomes (KEGG) were performed to identify which DEGs were significantly enriched in GO terms and metabolic pathways at Bonferroni-corrected *p*-value ≤ 0.05 compared with the whole-transcriptome background. GO functional enrichment and KEGG pathway analysis were carried out by Goatools (https://github.com/tanghaibao/Goatools accessed on 14 June 2021) and KOBAS 2.1.1 (http://kobas.cbi.pku.edu.cn/download.php accessed on 15 June 2021) [25].

### 2.13. Statistical Analysis

The statistical analyses were performed using SPSS 19.0 for Windows. Neurologic scores were expressed as median (range) and were compared using a nonparametric method (Kruskal–Wallis test) followed by the Mann–Whitney U statistic with Bonferroni correction. Except for the neurologic score, all data were expressed as mean ± standard deviation (S.D.) and were compared using a one-way analysis of variance (ANOVA) followed by Tukey’s multiple-comparison test. Values of *p* < 0.05 was considered statistically significant.

## 3. Results

### 3.1. Effects of Nuciferine on Anti-Cerebral Ischemia

**Figure 1:**
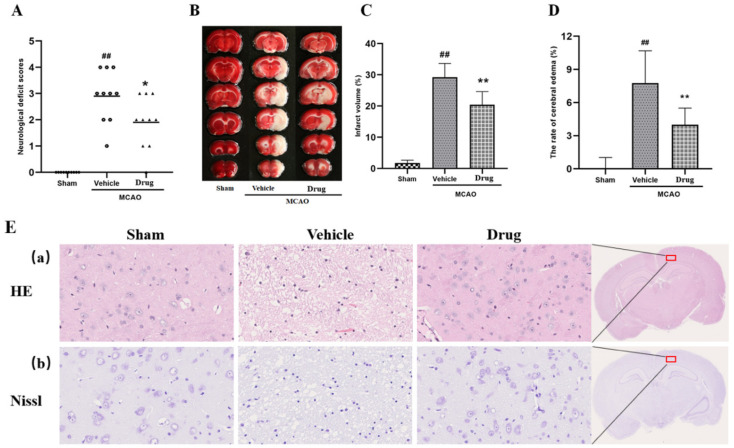
Nuciferine protected brain against middle cerebral artery occlusion. (**A**) Scatterplot of neurological deficit scores at 24 h after MCAO (sham, vehicle and drug group data were represented by squares, circles, and triangles, data underlined for medians, *n* = 10). (**B**) Representative photographs of TTC-stained coronal brain sections that show infarct regions tissue (white) 24 h after MCAO operation. (**C**) Statistical analyses of the effects produced by nuciferine on infarct volume in TTC-stained sections after MCAO injury (*n* = 10). (**D**) Quantitative analyses of rate of cerebral edema after MCAO injury (*n* = 10). Except for neurologic score, all data were expressed as mean ± SD; ## *p* < 0.01 compared with sham group; * *p* < 0.05, ** *p* < 0.01 compared with vehicle group. (**E**) Pathological results of brain injury in rats (**a**) HE staining, (**b**) Nissl staining.

To evaluate the protective effect of nuciferine on cerebral ischemia, the MCAO rats were established, and data from behavioural indicators and pharmacodynamics were obtained. Infarct volume, neurologic score and cerebral water content are the specific markers for evaluating brain injury. As shown in Figure 1A, there were no significant neurological deficits in the sham group, while severe neurological deficits were observed in the vehicle group. The treatment group with nuciferine showed significant improvement of the neurological deficits (*p* < 0.05 vs. vehicle group). For MCAO induced brain injury, which primarily manifested as cerebral infarct (Figure 1B,C), nuciferine treatment markedly reduced infarct volume from 29.23 to 20.44% (*p* < 0.01 vs. vehicle group). Furthermore, brain water content was remarkably increased in the ipsilateral hemisphere of rats in the vehicle group, which was significantly reduced by nuciferine treatment (*p* < 0.01, Figure 1D). These results suggested that nuciferine could protect against acute brain damage induced by cerebral ischemia.

### 3.2. HE Staining and Nissl Staining

The results of HE staining and Nissl staining were used to evaluate the protective effect of nuciferine on MCAO rats. The results showed that the brain tissue cells of the sham operation group had a clear outline, compact structure and normal cell morphology. The brain tissue of the vehicle group had vacuolar degeneration, sparse cell arrangement, fuzzy cell outline and structural disorder. The brain tissue of rats in the nuciferine treatment group was improved to some extent, which shows that nuciferine has a protective effect on brain tissue cells of rats with MCAO, as shown in Figure 1E.

### 3.3. Transcriptomic Analysis Results

#### 3.3.1. Sample Relationship Analysis

**Figure 2:**
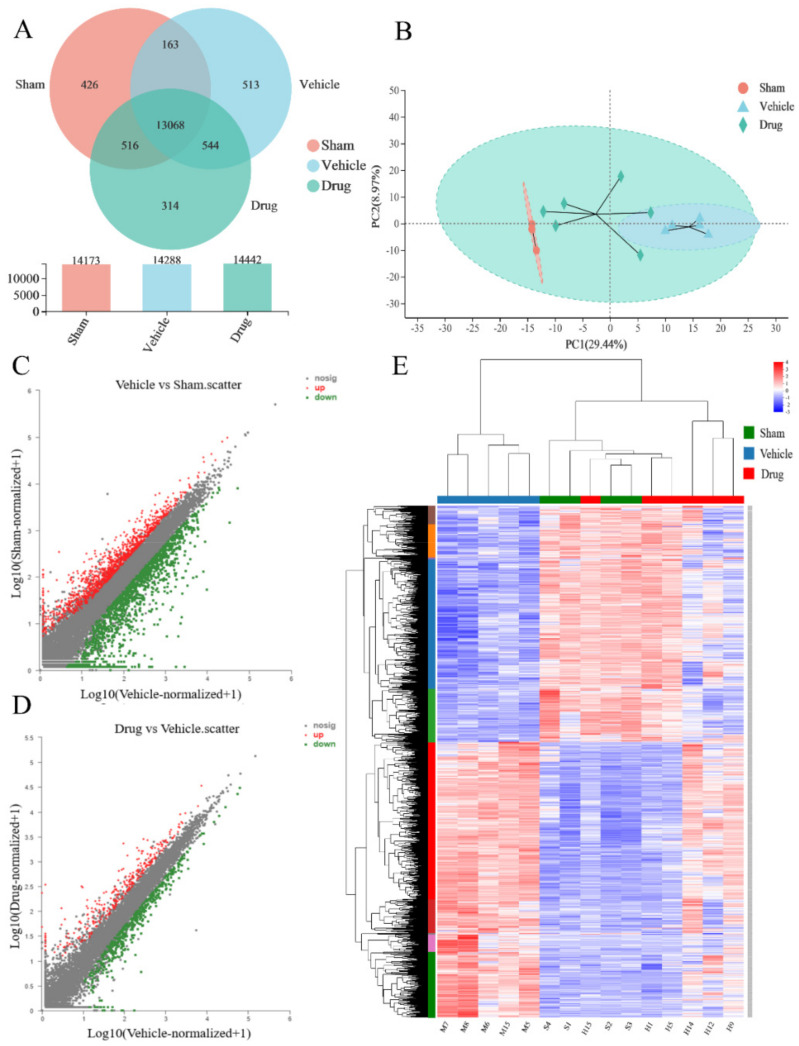
Results of sample relationship analysis and expression analysis. (**A**) Results of Veen analysis of the three groups of samples. (**B**) Results of principal component analysis of samples in three groups. (**C**) Scatter plot of gene expression in brain of rats in sham and vehicle group. (**D**) Scatter plot of gene expression in brain of rats in vehicle and drug group. (**E**) Results of cluster analysis of gene expression hierarchy of samples in three groups.

Veen analysis and PCA analysis were completed for common and unique genes between samples, based on gene expression. The Venn analysis identified genes shared or uniquely expressed between groups, and the results are shown in Figure 2A. PCA analysis, as an unsupervised method of data analysis, provides an overall picture of the variability between and within groups and a visualization of the trends and degrees of variation in the distribution of samples between groups. The analysis results showed that the sham group, the vehicle group and the drug (nuciferine) group could be clearly distinguished from each other. The brain tissue of rats in the vehicle group showed abnormal gene expression due to brain damage caused by ischemia, and that treatment with nuciferine could regulate gene expression in the brain of MCAO rats, shown in Figure 2B.

#### 3.3.2. Analysis of Sample Expression Differences

Gene expression in the brain tissue of three groups of rats was analyzed, and the scatter plot results showed that there were multiple differential genes in the brain tissue of the rats in the vehicle group compared with the sham group. Furthermore, there were multiple gene expression changes in the brain tissues of MCAO rats after treatment with nuciferine, as shown in Figure 2C,D. DESeq2 software was selected for analysis, and *p*-adjust < 0.05 and |log2FC| ≥ 2 were used for differential gene screening (fold change, FC). A total of 1919 differential genes were obtained for the vehicle group vs. the sham group, and 190 differential genes were obtained for the nuciferine group vs. the vehicle group. A total of 139 differential genes in the brain tissue of rats with drug-reversed MCAO were obtained by taking the intersection of the two differential genes, of which 69 were up-regulated and 70 were down-regulated, as shown in Table 1 (where only the top 10 genes in terms of fold change are listed). The results of hierarchical clustering analysis showed the reverse regulation of differential genes in the brain tissue of MCAO rats by nuciferine, as shown in Figure 2E.

#### 3.3.3. Functional Enrichment Analysis of Genes Regulated by Nuciferine

Functional enrichment analysis of the differential genes regulated by nuciferine, including GO enrichment and KEGG enrichment was performed. Fisher’s exact test was used, and when the corrected *p*-value (*p*-adjust) < 0.05 was used, the GO function or KEGG pathway was considered to be significantly enriched.

**Figure 3:**
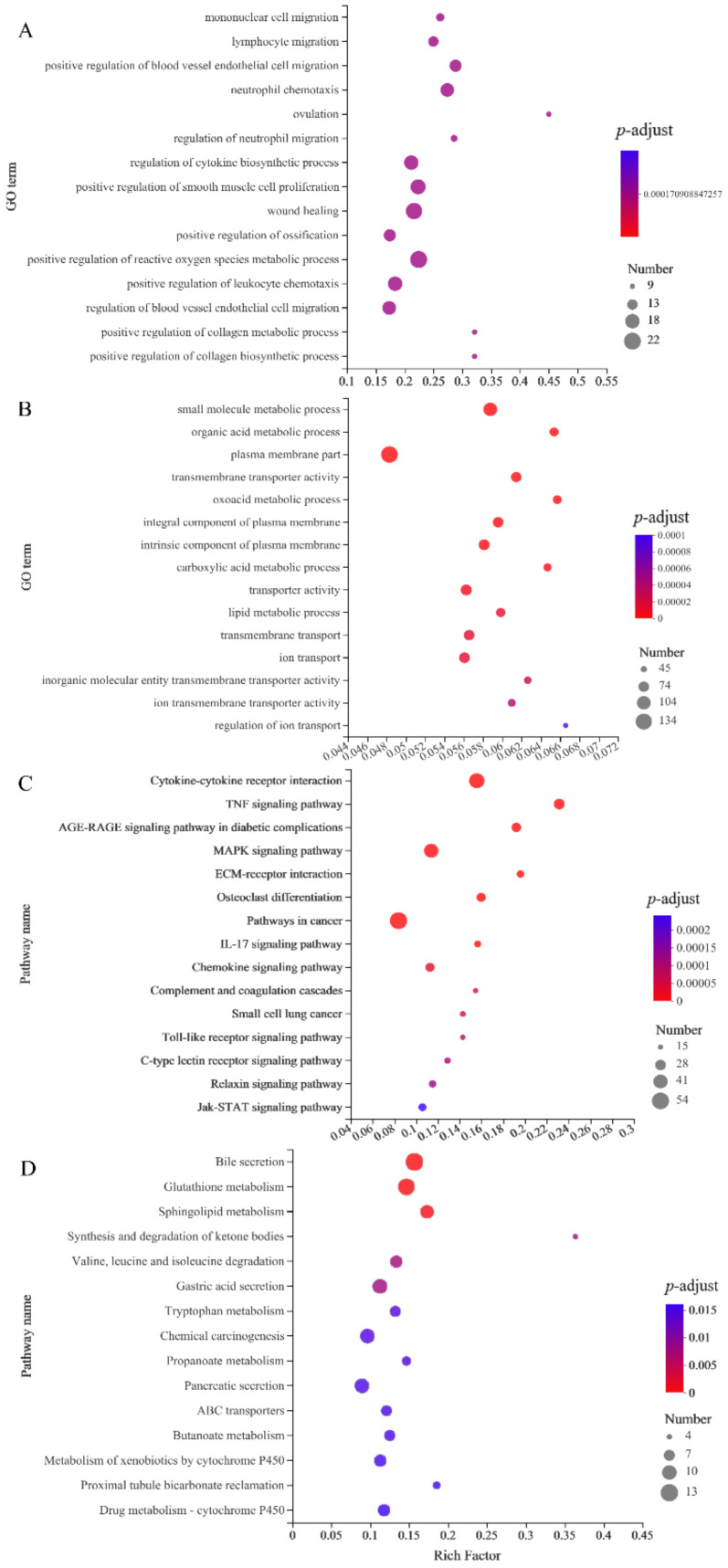
Results of GO and KEGG enrichment analysis of differential genes regulated by nuciferine. (**A**) GO enrichment analysis of up-regulated genes. (**B**) GO enrichment analysis of down-regulated genes. (**C**) KEGG enrichment analysis of up-regulated genes. (**D**) KEGG enrichment analysis of down-regulated genes. Only biological processes or pathways of TOP15 are shown in the figure.

GO function enrichment analysis showed that genes up-regulated by nucleotides were involved in the main biological processes, including monocyte migration, lymphocyte migration, positive regulation of vascular endothelial cell migration, neutrophil chemotaxis and regulation of neutrophil migration. The regulation of cytokine biosynthesis, wound healing, active oxygen metabolism, leukocyte chemotaxis, vascular endothelial cell migration, collagen biosynthesis and metabolism are shown in Figure 3A. The biological processes in which genes down-regulated by nuciferine are involved include small molecule metabolic process, organic acid metabolic process, oxoacid metabolic process, carboxylic acid metabolic process, lipid metabolic process and ion transport, as shown in Figure 3B.

The KEGG pathway enrichment analysis showed that differential genes involved in the upregulation of nuciferine included MAPK signaling pathway, Complement and coagulation cascades, Chemokine signaling pathway, IL-17 signaling pathway, Toll-like receptor signaling pathway and the Jak-STAT signaling pathway, as shown in Figure 3C. The pathways involved in the differential gene downregulation of nuciferine include glutathione metabolism, sphingolipid metabolism, synthesis and degradation of ketone bodies, valine, leucine and isoleucine degradation, tryptophan metabolism and butanoate metabolism, as shown in Figure 3D.

#### 3.3.4. Analysis of a Differential Gene Interaction Network Regulated by Nuciferine and Enrichment of KEGG Pathway

**Figure 4:**
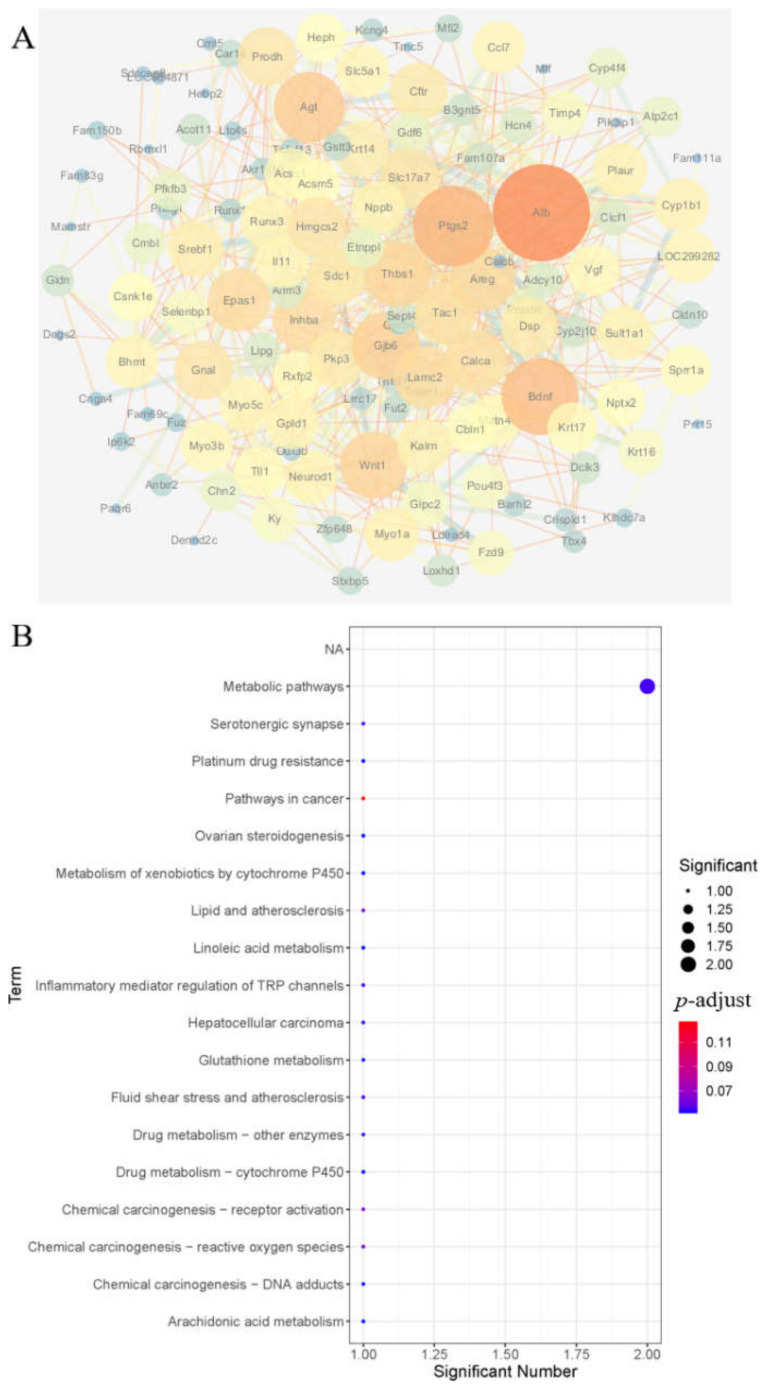
Protein interaction network diagram of key genes regulated by nuciferine and the enrichment results of the KEGG pathway. (**A**) Protein interaction network diagram, the nodes in the network represent proteins, the connecting lines indicates that there is an interaction between two proteins, and the larger the node the higher the degree of connectivity of the protein. (**B**) Enrichment results of the KEGG pathway of key genes.

Protein interaction network analysis of 139 differential genes (69 up-regulated and 70 down-regulated) regulated by nuciferine using the STRING 11.5 website, network analysis was performed using Cytoscape 3.7.1 software to calculate protein connectivity and to optimise the protein interactions network graph, as shown in Figure 4A.

The essential genes were screened according to having protein connectivity >5, and a total of 88 essential genes were identified for the MCAO rats treated with nuciferine, as shown in Table 2 (only the genes with top 20 connectivity were shown in the table). The KEGG pathway enriched the specific genes again. The results showed that the specific genes regulated by nuciferine were mainly involved in metabolic pathways, including lipid metabolic pathways (arachidonic acid metabolism, linoleic acid metabolism, sphingolipid metabolism) and PPARγ signaling pathways, amino acid metabolism, such as glutathione metabolism, and neural pathways, such as serotonergic synapses.

## 4. Discussion

MCAO can cause a series of secondary physiological and pathological changes in the brain. Acute cerebral ischemia leads to depolarisation of neurons, and causes acute injury mainly by neuronal osmotic edema, inducing neuronal necrosis and apoptosis [26]. Brain injury is influenced by a range of factors related to lipid changes, such as the activation of Ca^2+^ channels through the stimulation of excitatory amino acid receptors, and the massive inward flow of Ca^2+^ activates a range of Ca^2+^—dependent enzyme systems, including phosphodiesterase A2, phospholipase C and phospholipase D, causing delayed neuronal degeneration and necrosis. Phospholipase hydrolyses the phospholipid backbone of the cell membrane, such as phosphatidylcholine and phosphatidylethanolamine, into diglycerides, phosphatidic acid and arachidonic acid. Arachidonic acid produces phosphatidylglycerol, phosphatidylserine, phosphatidylinositol and phosphatidylethanolamine under the action of phospholipase for cell membrane reconstruction. In addition, under the action of cyclooxygenase and lipoxygenase, arachidonic acid produces inflammatory factors such as prostaglandins and leukotrienes and further induces neuronal apoptosis [27].

MCAO rats were prepared in this experiment by blocking the carotid artery, the blood flow could not be supplied immediately, which simulated the vascular embolism of clinical patients during a stroke. The results of brain histopathology showed that the brain tissue injury in the nuciferine treatment group was reduced, which showed that nuciferine had an apparent protective effect on the brain tissue of MCAO rats. According to the results of KEGG pathway enrichment for differential genes in rats treated with nuciferine, we observed that nuciferine protected brain tissue in rats with MCAO mainly involved in the regulation of critical genes such as Alb, Ptgs2, Bdnf, Agt, Gjb6, Wnt1, Thbs1, Areg, Lamc2, Inhba, Calca, Tac1, Hmgcs2, Gnal, Slc17a7, Cftr, Sdc1, Dsp, Pkp3, Srebf1, Krt14 and other vital genes, which when expressed regulated the lipid metabolism pathways such as arachidonic acid metabolism, sphingolipid metabolism and the PPARγ signaling pathway resulting in therapeutic effects. The specific explanations of the three pathways above are as follows.

**Figure 5:**
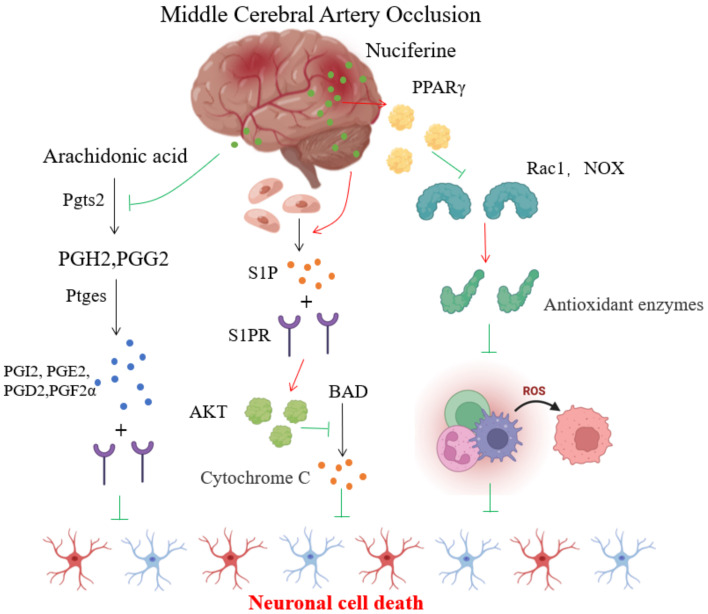
The protective mechanisms of nuciferine on MCAO rat brain.

Firstly, Arachidonic acid metabolism can be described as follows. The lipid metabolic pathway is a vital pathway for the therapeutic effect of nuciferine. Brain tissue contains a large number of lipids, lipids play an essential role in the evolution of the brain [28,29]. In MCAO, arachidonic acid is converted to arachidonic acid H2 (PGH2) and arachidonic acid G2 (PGG2) by prostaglandin G/H synthase 2 (Ptgs2), which is then metabolizsed by downstream prostaglandin synthase (Ptges) to produce various active prostaglandins, including PGI2, PGE2, PGD2, PGF2α, and a series of active prostaglandins that bind to specific receptors and mediate neuronal death in the brain. This experiment showed that nuciferine significantly down-regulated the gene expression of prostaglandin synthase 2, down-regulated the expression of a series of downstream prostaglandin synthase genes, reduced prostaglandin levels in the brain group and reduced neuronal death in brain tissue, as shown in Figure 5.

Secondly, according to sphingolipid metabolism, there is much to discuss further. The lipid metabolism pathway regulated by nuciferine also includes the sphingolipid metabolism pathway. Sphingolipids can be synthesizsed by all tissues in the body, with brain tissue being the most active. They are the main component of the membranes of nerve tissue. Phospholipases hydrolyse sphingolipids to produce ceramides, sphingomyelin and 1-phosphatidyl sphingomyelin (S1P), which are directly involved in regulating cell proliferation and apoptosis. Studies have shown that ceramide promotes apoptosis and inhibits cell growth and that S1P is a biologically active sphingolipid metabolite, with the brain being the organ with the highest concentration of S1P [30], and local S1P levels are further increased in MCAO [31]. It was found that in MCAO rats, S1P exerts a protective effect on cerebral ischemia through activation of S1Pl, which is associated with the activation of akt, which plays an essential role in the inhibition of apoptosis [32]. S1P can also induce the proliferation of neuronal cells [30], enhance the tolerance of cortical neurons and endothelial cells to ischemia and hypoxia and counteract ischemia-hypoxia-induced brain cell death. Inhibition of S1P signaling pathway can prevent post-ischemic blood–brain barrier dysfunction [33].

Sphingosine kinases (Sph Ks) are the key enzymes that catalyse the production of S1P from sphingosine and are essential for regulating S1P levels. The regulation of S1P levels by sphingosine kinase is also dependent on the levels of S1P precursors—sphingosine and ceramide. The two main isomers, Sph K1 and Sph K2, are mainly located in the cerebral cortex and hippocampus, and studies have shown that increasing the expression activity and activity of Sph K2 reduces infarct size and brain edema [34]. In this experiment, the genes significantly up-regulated by nuciferine were involved in sphingolipid metabolism. A total of nine up-regulated genes were enriched in this pathway, suggesting that nuciferine significantly up-regulates gene expression of various enzymes and reduces cell death and infarct size in brain tissue, as shown in Figure 5.

Thirdly, the PPARγ signaling pathway is also something worth studying deeply. The results showed that the level of PPARγ in the brain of MCAO rats was significantly reduced and was significantly up-regulated in the brain of rats treated with nuciferine. PPARγ is distributed in multiple brain regions, binds to specific DNA response elements, regulates gene transcription and expression and modulates multiple physiological functions [35]. Studies have shown that PPARγ reduces glutamate toxicity [36] and calcium overload, decreases oxidative stress [37] and endoplasmic reticulum stress [38] in the MCAO and exerts anti-inflammatory [39] and apoptosis-reducing [40] effects. PPARγ reduces cell damage by inhibiting the activity of ras-related C3 botulinum toxin substrate l (Rac1) and nicotinamide adenine dinucleotide phosphate (NADPH) oxidase (NOX), increasing the expression of antioxidant enzymes and reducing ROS production. This study showed that nuciferine significantly reduced the expression of Rac1 and NOX genes, suggesting that rhodopsin could play a protective role in brain tissue by upregulating PPARγ activity and reducing the expression of Rac1 and NOX genes to combat oxidative stress and reduce cell damage, shown in Figure 5.

## 5. Conclusions

In a nutshell, the present findings suggest that nuciferine could reduce the production of inflammatory factors in the brain of MCAO rats, resist oxidative stress and decrease the amount of cell death and infarct size in brain tissue by modulating lipid metabolic pathways, which contain regulation of arachidonic acid metabolism, sphingolipid metabolism and the PPARγ signaling pathway obtaining gene expression of key enzymes. This research suggests that nuciferine treatment is an attractive option for targeting MCAO.

## Figures and Tables

**Table 1:** Up- and down-regulated genes in brain of MCAO rats regulated by nuciferine.

Transcript ID	Gene ID	Gene Name	Vehicle vs. Sham	Drug vs. Vehicle	Fold Change
ENSRNOT00000045087	ENSRNOG00000013305	*Atp2c1*	down	up	1,682,348.77
ENSRNOT00000040559	ENSRNOG00000013351	*Stxbp5*	down	up	169.688
ENSRNOT00000081050	ENSRNOG00000018716	*Dennd2c*	down	up	168.795
ENSRNOT00000076780	ENSRNOG00000011160	*AC126641.1*	down	up	153.802
ENSRNOT00000025172	ENSRNOG00000010440	*Gnal*	down	up	101.410
ENSRNOT00000016184	ENSRNOG00000012138	*Rbmxl1*	down	up	100.251
ENSRNOT00000025465	ENSRNOG00000018842	*Pou4f3*	down	up	39.891
ENSRNOT00000005382	ENSRNOG00000026371	*Krt17*	down	up	35.125
ENSRNOT00000019477	ENSRNOG00000014441	*Krt16*	down	up	26.506
ENSRNOT00000002887	ENSRNOG00000002117	*Barhl2*	down	up	26.208
ENSRNOT00000088424	ENSRNOG00000013076	*Csnk1e*	up	down	0.000
ENSRNOT00000092103	ENSRNOG00000012294	*Heph*	up	down	0.003
ENSRNOT00000089142	ENSRNOG00000015428	*Mff*	up	down	0.008
ENSRNOT00000083336	ENSRNOG00000053084	*Fuz*	up	down	0.010
ENSRNOT00000012655	ENSRNOG00000009411	*Chn2*	up	down	0.024
ENSRNOT00000011742	ENSRNOG00000022116	*Gjb6*	up	down	0.065
ENSRNOT00000001222	ENSRNOG00000000920	*Phkg1*	up	down	0.073
ENSRNOT00000048453	ENSRNOG00000028837	*AC128207.1*	up	down	0.086
ENSRNOT00000015855	ENSRNOG00000011716	*Degs2*	up	down	0.105
ENSRNOT00000021120	ENSRNOG00000015768	*Nat8f5*	up	down	0.111

Note: Only the top 10 up- and down-regulated genes are shown.

**Table 2:** Key genes regulated by nuciferine treatment in MCAO rats.

Transcript ID	Gene ID	Gene Name	Degree	Betweenness Centrality	Closeness Centrality
ENSRNOT00000003921	ENSRNOG00000002911	*Alb*	48	0.1530	0.5829
ENSRNOT00000003567	ENSRNOG00000002525	*Ptgs2*	35	0.0669	0.5256
ENSRNOT00000083542	ENSRNOG00000047466	*Bdnf*	32	0.0757	0.5279
ENSRNOT00000024917	ENSRNOG00000018445	*Agt*	27	0.0360	0.5062
ENSRNOT00000011742	ENSRNOG00000022116	*Gjb6*	25	0.0361	0.4862
ENSRNOT00000090156	ENSRNOG00000061818	*Wnt1*	24	0.0450	0.5000
ENSRNOT00000070912	ENSRNOG00000045829	*Thbs1*	23	0.0142	0.4843
ENSRNOT00000003703	ENSRNOG00000002754	*Areg*	22	0.0312	0.4767
ENSRNOT00000036947	ENSRNOG00000002667	*Lamc2*	21	0.0226	0.4659
ENSRNOT00000019272	ENSRNOG00000014320	*Inhba*	21	0.0390	0.4862
ENSRNOT00000014948	ENSRNOG00000011130	*Calca*	20	0.0218	0.4862
ENSRNOT00000034719	ENSRNOG00000007374	*Tac1*	20	0.0331	0.4731
ENSRNOT00000026121	ENSRNOG00000019120	*Hmgcs2*	18	0.0211	0.4556
ENSRNOT00000025172	ENSRNOG00000010440	*Gnal*	18	0.0657	0.4731
ENSRNOT00000092245	ENSRNOG00000020650	*Slc17a7*	18	0.0239	0.4731
ENSRNOT00000085043	ENSRNOG00000055103	*Cftr*	17	0.0239	0.4624
ENSRNOT00000086633	ENSRNOG00000059947	*Sdc1*	17	0.0132	0.4624
ENSRNOT00000018649	ENSRNOG00000013928	*Dsp*	16	0.0082	0.4539
ENSRNOT00000020864	ENSRNOG00000015152	*Pkp3*	16	0.0262	0.4362
ENSRNOT00000004753	ENSRNOG00000003463	*Srebf1*	15	0.0450	0.4659

Note: Only the top 20 genes in terms of connectivity are shown.

## Data Availability

Data are available on request from the corresponding author.
